# A muti-informant national survey on the impact of COVID-19 on mental health symptoms of parent–child dyads in Canada

**DOI:** 10.1038/s41598-023-34544-7

**Published:** 2023-05-17

**Authors:** Jeanna Parsons Leigh, Stephana Julia Moss, Cynthia Sriskandarajah, Eric McArthur, Sofia B. Ahmed, Kathryn Birnie, Donna Halperin, Scott Halperin, Micaela Harley, Jia Hu, Josh Ng Kamstra, Laura Leppan, Angie Nickel, Nicole Racine, Kristine Russell, Stacie Smith, May Solis, Maia Stelfox, Perri R. Tutelman, Henry T. Stelfox, Kirsten M. Fiest

**Affiliations:** 1 Faculty of Health, Dalhousie University, Halifax, NS Canada; 2 London Health Sciences Centre, London, ON Canada; 3 Department of Medicine, University of Calgary, Calgary, AB Canada; 4 Department of Anesthesiology, Perioperative, and Pain Medicine, Calgary, Canada; 5 Department of Community Health Sciences, University of Calgary, Calgary, AB Canada; 6 Rankin School of Nursing, St. Francis Xavier University, Antigonish, NS Canada; 7 Faculty of Medicine, Dalhousie University, Halifax, NS Canada; 8 Frayme, Cornwall, ON Canada; 9 Department of Surgery, University of Hawaii John A Burns School of Medicine, Honolulu, HI Canada; 10 Faculty of Social Sciences, University of Ottawa, Ottawa, ON Canada; 11 Sepsis Canada, Hamilton, ON Canada; 12 Young Canadian Roundtable On Health, Toronto, ON Canada; 13 Department of Oncology, University of Calgary, Calgary, Canada; 14 Department of Critical Care Medicine, Calgary, Canada; 15 O’Brien Institute for Public Health, University of Calgary, Calgary, Canada

**Keywords:** Health care, Risk factors

## Abstract

The COVID-19 pandemic negatively impacted the mental health of children, youth, and their families which must be addressed and prevented in future public health crises. Our objective was to measure how self-reported mental health symptoms of children/youth and their parents evolved during COVID-19 and to identify associated factors for children/youth and their parents including sources accessed for information on mental health. We conducted a nationally representative, multi-informant cross-sectional survey administered online to collect data from April to May 2022 across 10 Canadian provinces among dyads of children (11–14 years) or youth (15–18 years) and a parent (> 18 years). Self-report questions on mental health were based on The Partnership for Maternal, Newborn & Child Health and the World Health Organization of the United Nations H6+ Technical Working Group on Adolescent Health and Well-Being consensus framework and the Coronavirus Health and Impact Survey. McNemar’s test and the test of homogeneity of stratum effects were used to assess differences between children-parent and youth-parent dyads, and interaction by stratification factors, respectively. Among 933 dyads (N = 1866), 349 (37.4%) parents were aged 35–44 years and 485 (52.0%) parents were women; 227 (47.0%) children and 204 (45.3%) youth were girls; 174 (18.6%) dyads had resided in Canada < 10 years. Anxiety and irritability were reported most frequently among child (44, 9.1%; 37, 7.7%) and parent (82, 17.0%; 67, 13.9%) dyads, as well as among youth (44, 9.8%; 35, 7.8%) and parent (68, 15.1%; 49, 10.9%) dyads; children and youth were significantly less likely to report worsened anxiety (*p* < 0.001, *p* = 0.006, respectively) or inattention (*p* < 0.001, *p* = 0.028, respectively) compared to parents. Dyads who reported financial or housing instability or identified as living with a disability more frequently reported worsened mental health. Children (96, 57.1%), youth (113, 62.5%), and their parents (253, 62.5%; 239, 62.6%, respectively) most frequently accessed the internet for mental health information. This cross-national survey contextualizes pandemic-related changes to self-reported mental health symptoms of children, youth, and families.

## Introduction

Children and youth have felt substantial mental health impacts of the COVID-19 pandemic^1,2^. They have experienced uncertainties, considerable changes to routines, fears, and social and physical isolation in parallel to high levels of parental stress regarding threats to their family’s health and economic welfare^1–6^. The information and communication challenges associated with the distribution and uptake of public health evidence and recommendations throughout the COVID-19 pandemic have been well documented^7–13^, as have the continued associated devasting effects on mental health^3,14–18^. Prioritizing broad mental health, including child and youth mental health, is an essential component of any pandemic response effort and future pandemic preparedness planning.

The response of a child or youth to an emergency situation depends on their previous exposure to emergencies as well as their physical and mental health, cultural background, and the socio-economic circumstances of the family^19–21^. Studies have shown that crisis events have differential negative impacts on the mental health of children and youth, compared to their parents^22–25^, resulting in increased emotional stress, feelings of helplessness, and fear that can evolve into mental health diagnoses such as anxiety, depressive, or posttraumatic stress disorders^26,27^. Research early in the pandemic identified social-economic stability as a common contributing factor to the unequal distribution of negative mental health impacts among children and youth and their families^28–32^. Mental health strategies that consider the differential evolution of mental health symptoms and associated factors between children/youth and their parents and are adaptable to individualized child, youth, and family needs are required^33^.

The objective of this work was to conduct a large, nationally representative, multi-informant cross-sectional survey to measure how mental health symptoms of children, youth, and their parents changed during the COVID-19 pandemic and to identify factors associated with symptomatic changes including the sources accessed for information related to mental health. This research can inform the development and delivery of evidence-based and demographic-tailored mental health interventions for children and youth considering a broad range of available healthcare resources and parental capacities.

## Methods

### Study design and population

We collected data from an anonymous, voluntary, 10-min cross-sectional survey via Leger, a Canadian-based market research and polling firm (https://leger360.com), between April 20, 2022, and May 25, 2022, which was a time period when most Canadian provinces had eased social restrictions^34^. We used Leger’s dynamic Leger Opinion (LEO) panel, an online pool of over 400,000 individuals recruited and validated through multiple methods who consented to be contacted for research purposes; at any given time this pool reflects a representative sample of Canadian residents with internet access. Respondents received LEO reward points after completing the questionnaire, which could be redeemed for gift cards and merchandise. Assuming children and youth aged 11–18 represent ~ 11% of the Canadian population (~ 4 million)^35^ we recruited 1600 respondents (800 dyads (i.e., a group of two members)) to conduct subgroup analyses with a ± 3.5% margin of error at a 95% confidence level, and required that at least 15% of the sample included dyads who had lived in Canada for less than 10 years (5% less than 5 years) in order to understand their unique experiences. The (total) 85-item (English and French) electronic survey was administered to LEO panelists who identified as parents or legal guardians (> 18 years of age; hereafter referred to as parents) with at least one child (11–14 years of age) or youth (15–18 years of age) living in the same household; the oldest child or youth was selected if more than one was eligible. Age ranges for children and youth were selected to align with Statistics Canada standards and to adhere to institutional ethical requirements (e.g., age-tailored questions)^36^. Parents first completed their portion (45-items) independently followed by their child or youth (40-items), who completed their own portion independently. We followed the Checklist for Reporting Results of Internet E-Surveys (CHERRIES) guidelines (Supplemental Table 1)^37^.

**Table 1:** Demographics and Characteristics of 933 Child/Youth-Parent Dyad Survey Participants in the COVID-19 Pandemic.

Characteristic	Child (11–14 y)	Youth (15–18 y)	Parents (> 18 y)
	Value, No. (%)	Value, No. (%)	Value, No. (%)
	N = 483	N = 450	N = 933
Sex			
Male	239 (49.5)	235 (52.2)	447 (47.9)
Female	239 (49.5)	214 (47.6)	486 (52.1)
Prefer not to answer	5 (1.0)	1 (0.2)	0 (0.0)
Gender			
Woman	227 (47.0)	204 (45.3)	485 (52.0)
Man	242 (50.1)	226 (50.2)	415 (44.5)
Non-binary	2 (0.4)	6 (1.3)	6 (0.7)
Two-Spirit	1 (0.1)	2 (0.4)	1 (0.1)
Prefer not to answer	2 (0.4)	7 (1.6)	26 (2.8)
Age, years			
11–14	100 (100.0)	0 (0.0)	0 (0.0)
15–18	0 (0.0)	100 (100.0)	0 (0.0)
19–24	0 (0.0)	0 (0.0)	35 (3.8)
25–34	0 (0.0)	0 (0.0)	88 (9.4)
35–44	0 (0.0)	0 (0.0)	349 (37.4)
45–54	0 (0.0)	0 (0.0)	335 (35.9)
55–64	0 (0.0)	0 (0.0)	113 (12.1)
65–74	0 (0.0)	0 (0.0)	12 (1.3)
≥75	0 (0.0)	0 (0.0)	1 (0.1)
Disability			
Yes – visible	11 (2.3)	11 (2.4)	38 (4.1)
Yes – invisible	31 (6.4)	23 (5.1)	83 (8.9)
No	439 (90.9)	414 (92.0)	806 (86.4)
Ethnicity			
Black, Indigenous, and People of color	134 (27.7)	134 (29.8)	266 (28.5)
White	335 (69.4)	317 (70.4)	654 (70.1)
Prefer to self-describe	14 (2.9)	5 (0.01)	13 (1.4)
Geographic location			
Atlantic (BF, NB, NS, PEI)	N/A	N/A	41 (4.4)
Central (QC, ON)			654 (70.1)
Prairies (MB, SK, AB)			139 (14.9)
West coast (BC)			99 (10.6)
Size of household			
< 4	N/A	N/A	698 (74.8)
≥5			232 (24.9)
Canadian residence, years			
< 1	N/A	N/A	6 (0.6)
1–4			37 (4.0)
5–9			131 (14.0)
10–19			80 (8.6)
≥20			678 (72.7)
Self-rated COVID-19 knowledge			
Very poor	19 (3.9)	12 (2.7)	63 (6.8)
Poor	62 (12.8)	64 (14.2)	166 (17.8)
Average	252 (52.2)	210 (46.7)	198 (21.2)
Good	129 (26.7)	111 (24.7)	256 (27.4)
Very good	21 (4.4)	52 (11.6)	249 (26.7)
Previously diagnosed with COVID-19			
Yes	172 (35.6)	141 (31.3)	293 (31.4)
No	310 (64.2)	306 (68.0)	640 (68.6)
Job loss during COVID-19			
Yes	N/A	114 (25.3)	97 (10.4)
No		138 (30.7)	703 (73.4)
Not applicable		198 (44.0)	133 (14.3)
Social media use per day, hours			
None	99 (20.5)	23 (5.1)	76 (8.2)
< 1	122 (25.3)	95 (21.1)	291 (21.3)
1–3	155 (32.1)	198 (44.0)	356 (38.2)
4–6	83 (17.2)	90 (20.0)	109 (11.7)
> 6	24 (5.0)	44 (9.8)	101 (10.8)

AB, Alberta; BC, British Columbia; MB, Manitoba; N/A, Not Asked; NB New Brunswick; NS, Nova Scotia; ON, Ontario; PEI, Prince Edward Island; QC, Quebec; SK, Saskatchewan.

### Survey development

We created a preliminary list of mental health questions based on findings presented in published articles identified in our scoping review^38^ and systematic review^39^ on strategies, approaches, and interventions to improve youth wellbeing during the COVID-19 pandemic and mapped them onto The Partnership for Maternal, Newborn & Child Health and the World Health Organization of the United Nations H6 + Technical Working Group on Adolescent Health and Well-Being consensus framework for defining, programming, and measuring adolescent wellbeing that is part of a broader program of work that includes a multistakeholder Call to Action to prioritize adolescent well-being. This framework includes five domains: (1) Good health and optimum nutrition; (2) Connectedness, positive values, and contribution to society; (3) Safety and a supportive environment; (4) Learning, competence, education, skills, and employability; and (5) Agency and resilience (Supplemental Table 2)^40^. Operational definitions for self-reported mental health symptoms are provided in Supplemental Table 3. Demographic, knowledge acquisition, and health literacy questions were based on the Coronavirus Health and Impact Survey (CRISIS)^41^. We developed a combination of continuous, categorial, Likert-type, and open-ended response options; Likert-type questions included a scale ranging from 1 (i.e., “a little”) to 5 (i.e., “a lot”). Questions were iteratively refined by the core survey development team (JPL, SJM, RBM, DH, SH, PT)^42^ and six citizen partners (three youth: MS, MH, SS, and three parents: KR, MS, AN). The order of the response options was randomized and attention checks (innocuous questions with a single correct answer) were randomly inserted throughout the questionnaire. One question was presented per screen and respondents were not given the opportunity to change their answers once they moved to the next screen; all questions included a “don’t know” or “prefer not to answer” option that were excluded from analyses.

**Table 2:** Mental Health Knowledge Acquisition by Source of 933 Child/Youth-Parent Dyad Survey Participants in the COVID-19 Pandemic.

Domain	Response	Child N = 483		Parent N = 483		*p*-value	Youth N = 450		Parent N = 450		*p*-value
Sourced information	Yes	168	34.8%	405	83.9%	< 0.001	216	48.0%	382	84.9%	< 0.001
Information source^a^	The internet	96	57.1%	253	62.5%	0.16	113	62.5%	239	62.6%	0.04
	The television	45	26.8%	187	46.2%	< 0.001	64	46.2%	180	47.1%	< 0.001
	A healthcare professional	46	27.4%	216	53.3%	< 0.001	45	53.3%	174	45.5%	< 0.001
	A newspaper or magazine	21	12.5%	139	34.3%	< 0.001	31	34.3%	118	30.9%	< 0.001
	A social media platform	54	32.1%	122	30.1%	0.60	65	30.1%	96	25.1%	0.67
Source influences	Reliability of information	78	46.4%	266	65.7%	< 0.001	106	65.7%	254	66.5%	< 0.001
	User friendly format	60	35.7%	102	25.2%		71	25.2%	104	27.2%	
	Recommendation from friend	20	11.9%	18	4.4%		23	4.4%	13	3.4%	
	Confidentiality of information	3	1.8%	2	0.5%		4	0.5%	3	0.8%	
	Low cost	2	1.2%	5	1.2%		3	1.2%	1	0.3%	
Experienced difficulties	Yes	36	21.4%	77	19.0%	0.11	34	19.0%	62	16.2%	0.13
Source preferences^a^	A healthcare professional	78	46.4%	232	57.3%	< 0.001	87	57.3%	205	53.7%	< 0.001
	The internet	65	38.7%	171	42.2%	0.02	82	42.2%	163	42.7%	0.33
	The television	45	26.8%	150	37.0%	< 0.001	44	37.0%	137	35.9%	< 0.001
	A newspaper or magazine	18	10.7%	82	20.2%	< 0.001	14	20.2%	52	13.6%	0.01

^a^Response options not mutually exclusive; *p*-values represent individual (within dyad) comparisons. Respondents who did not provide an answer were excluded from analyses.

### Statistical analysis

Continuous variables were summarized using mean (standard deviation, SD) or median (interquartile range, IQR), as appropriate. Categorical variables were presented as frequency (percentage). Survey question responses were compared between parents and their children. For ordinal Likert-type questions, the Wilcoxon signed-rank test was used as it accounts for the paired nature of the data and the correlation present between parent and child^43^. Responses to nominal questions were compared between parent and child using McNemar’s test for paired data^44^. In subgroup analyses, responses were stratified by prespecified (e.g., gender, geographic location, ethnicity) demographic characteristics. For the nominal questions, potential interaction by the stratification factors was assessed using a test of homogeneity of stratum effects, an extension of McNemar’s test^45^. For clarity of presentation, “a little” or “a lot” better or worse are presented in aggregate as “better” or “worse." Two-sided *p*-values < 0.05 were considered statistically significant. All analyses were conducted using R version 4.2.1^46^.

### Patient and public involvement

We abided by the Canadian Institutes of Health Research (CIHR)-guiding core principles of inclusiveness, mutual respect, support, and co-building^47^ and adhered to the GRIPP-2 reporting guidelines for patient and public involvement^48^. Youth and parent involvement in the current project began in 2021; they participated in group discussion alongside other stakeholders (e.g., researchers, clinicians, decision makers). The research questions, protocol, and this paper were jointly developed with youth (MS, SS, MH) and parent (AN, MS, KR) partners on this team. All youth and family partners are compensated for their time.

### Ethical considerations

All participants provided electronic informed consent on their own behalf; as the parent had significant knowledge of their child/youth, prior to submitting their own consent, the parent attested that they understood the information regarding their child/youth’s participation and that their child/youth had the capacity to consent on their own behalf. This study was approved by the University of Calgary’s Conjoint Health Research Ethics Board (#21–2013) and the Research Ethics Board at Dalhousie University (#2021–5947); all methods were carried out according to guidelines and regulations.

### Ethical approval

This study was approved by the University of Calgary’s Conjoint Health Research Ethics Board (#21–2013) and the Research Ethics Board at Dalhousie University (#2021–5947).

## Results

### Survey participants

Among the 933 dyads (N = 1866 individual respondents), 483 (51.7%) included children (aged 11–14 years; 227, 47.0% girls), and 450 (48.3%) included youth (aged 15–18 years; 204, 45.3% girls); parents were most frequently aged 35–44 years (349, 37.4%) or 45–54 years (335, 35.9%) and 415 (44.5%) parents were women (Table 1). Most families (698, 74.8%) consisted of four or fewer members and had resided in Canada for over 20 years (678, 72.7%); 211 (22.6%) families had resided in Canada between five and 10 years.

### Self-reported mental health symptoms

#### Prevalence

**Figure 1:**
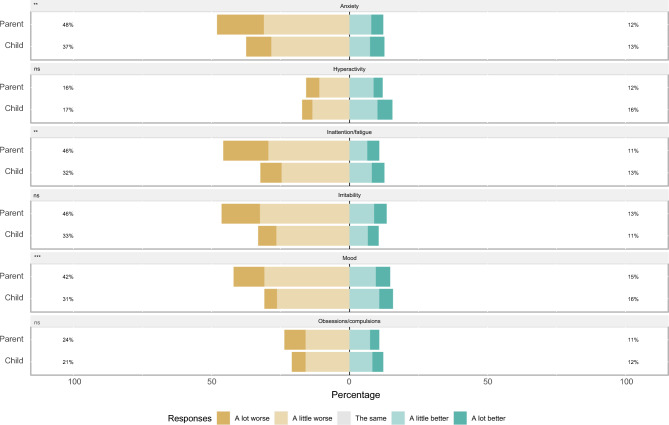
Mental Health Impacts of 483 *Child*-Parent Dyad Survey Participants in the COVID-19 Pandemic**.** Respondents were asked, “Compared to the time before the COVID-19 pandemic, how is your [mental health domain].” *P*-values obtained using Wilcoxon signed rank test, taking into the paired nature of the comparison. This means 483 parents of children were compared to their children. Respondents who did not provide an answer were excluded from analyses. *, *p* < 0.05; **, *p* < 0.01; ***, *p* < 0.001; ns, not statistically significant.

Anxiety, irritability, and inattention were the areas of mental health that children (37.5%, 33.1%, 32.3%, respectively), youth (37.1%, 35.6%, 32.4%, respectively), and their parents (of children, 48.1%, 46.4%, 45.8%, and of youth, 45.3%, 37.8%, 37.1%, respectively) most frequently reported had worsened during the COVID-19 pandemic (Fig. 1 and Supplemental Table 4). Compared to their parents, children less frequently reported worsened mood (*p* < 0.001), anxiety (*p* < 0.001), irritability (*p* < 0.001), and inattention (*p* < 0.001) during the COVID-19 pandemic, while youth less frequently reported worsened mood (*p* < 0.001), anxiety (*p* = 0.006), and inattention (*p* = 0.028) during the COVID-19 pandemic.

#### Stratified analyses

Children, youth, and their parents who experienced financial instability (561 dyads, 60.1%) compared to dyads who did not experience financial instability during the COVID-19 pandemic more frequently reported worse mental health. Specifically they reported heightened symptoms of anxiety (child 42% and parent 55%, youth 43% and parent 54%), irritability (child 37% and parent 54%, youth 45% and parent 48%), and inattention (child 38% and parent 53%, youth 41% and parent 46%) (Supplemental Fig. 1). Similarly, children, youth, and their parents who experienced housing instability (542 dyads, 58.1%) compared to dyads who did not experience housing instability during the COVID-19 pandemic more frequently reported worse mental health including symptoms of anxiety (child 45% and parent 57%, youth 42% and parent 55%), irritability (child 40% and parent 56%, youth 44% and parent 48%), and inattention (child 40% and parent 55%, youth 39% and parent 46%) (Supplemental Fig. 2). Children, youth, and their parents who self-identified as a person living with a disability compared to dyads who did not self-identify as living with a disability more frequently reported worse mental health that was heightened for symptoms of anxiety (child 43% and parent 55%, youth 52% and parent 53%), irritability (child 43% and parent 57%, youth 56% and parent 51%), and inattention (child 39% and parent 48%, youth 45% and parent 52%) (Supplemental Fig. 3). Stratified analyses by ethnicity, gender, geographical location, employment status, and household size are provided in Supplemental Figs. 4 through 9.

**Figure 2:**
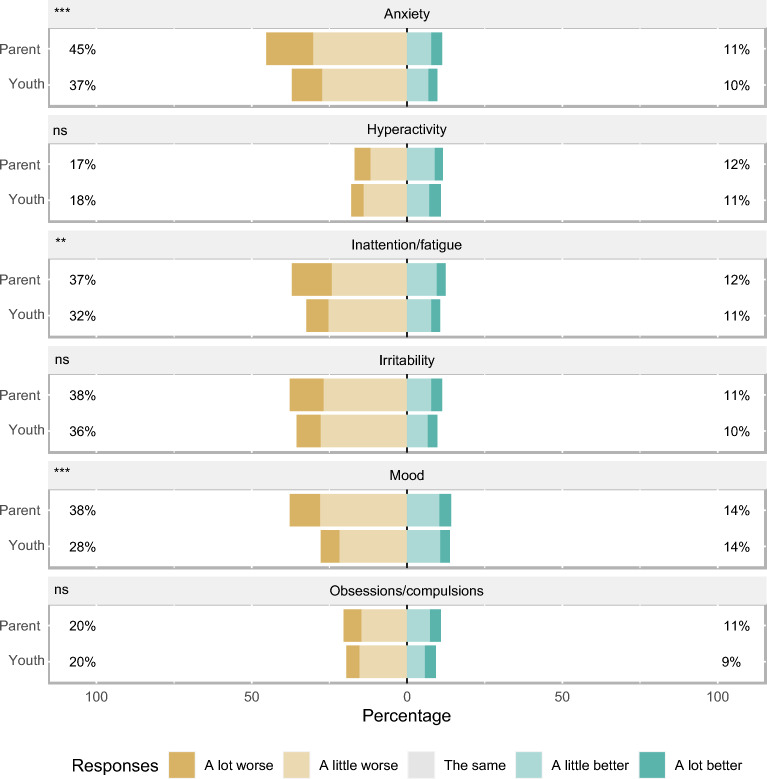
Mental Health Impacts of 450 *Youth*-Parent Dyad Survey Participants in the COVID-19 Pandemic. Respondents were asked, “Compared to the time before the COVID-19 pandemic, how is your [mental health domain].” *P*-values obtained using Wilcoxon signed rank test, taking into the paired nature of the comparison. This means 450 parents of youth were compared to their youth. Respondents who did not provide an answer were excluded from analyses. *, *p* < 0.05; **, *p* < 0.01; ***, *p* < 0.001; ns, not statistically significant.

#### Information accessed

Compared to their parents, significantly fewer children (34.8 vs. 83.0%, *p* < 0.001) and youth (48.0 vs. 84.9%, *p* < 0.001) accessed (any) information on mental health during the COVID-19 pandemic (Table 1). Among those who did, the internet was the most frequently accessed source of mental health information (child 57.1% and parent 62.5%, youth 62.5% and parent 62.6%) (Table 2). Healthcare professionals were most frequently reported as the preferred source of mental health information on among children (46.4%), youth (57.3%), and parents (57.3% and 53.7%, respectively). Across all categories children and youth were significantly less confident regarding their knowledge acquisition abilities, including vetting of the information, when compared to their parents (Fig. 2, Supplemental Table 5).

## Discussion

In this nationally representative, multi-informant survey we identified that over one-third of children, youth, and their parents self-reported that their mental health symptoms had worsened during the COVID-19 pandemic. We also found that children and youth were significantly less likely to self-report worsened mental health symptoms compared to their parents. Children and youth, compared to their parents, were significantly less confident in their ability to verify and understand information on mental health that was most frequently accessed via the internet.

Children in our sample reported worsened symptoms of anxiety, inattention, and irritability while youth in our sample reported worsened symptoms of anxiety, inattention, and mood. These findings highlight developmental differences with regards to symptom presentation associated with the COVID-19 pandemic. Interestingly, children and youth presented with similar exacerbation in symptoms, including worsened symptoms of anxiety and inattention. In younger children, mood challenges often present as increased irritability, whereas low and depressed mood are symptoms that tend to emerge during adolescence and early to mid-adulthood^49,50^. Further, psychological adaptation matters in the context of our study, because we surveyed participants two years after the very early phase of the pandemic (May 2022 compared to May 2020), when short-lived changes in mental health symptoms had potentially diminished. Although mental health symptoms typically return to baseline levels following common life experiences^51,52^, the COVID-19 pandemic may have had different effects. Specifically, COVID-19 was unique in scale and social and economic consequences, and it is therefore likely that a portion of the population will experience ongoing mental health difficulties. It will be important to identify and support children and youth who are most vulnerable^53–55^.

Parents reported significantly worse mental health symptoms compared to their children and youth. Loss of routine occurred rapidly for many families during the COVID-19 pandemic related to changes in employment arrangements, school closures, and loss of access to activities outside of the home such as sports and extracurriculars^56^. Several studies have identified that children and youth in families that maintained a structured routine demonstrated lower rates of externalizing problems, over and above the effect of food insecurity, socioeconomic status, dual-parent status, maternal depression, and stress^57,58^; these findings are consistent with prior work suggesting that lack of predictability is linked strongly to youth psychopathology^59–62^. However, maintaining routine and structure for parents was challenging as school closures were unpredictable and impacted many aspects of daily life and may have come at a cost to their own mental health^63^. The consequences of these difficulties were likely longstanding, related perhaps to the ways in which contextual risk permeates the structures and processes of family systems^64^. Our findings suggest that adequate mental health resources for parents are required as one component in the cascading process involving parental mental health and family processes for families to fully reap potential benefits associated with maintaining routine for children and youth during a pandemic.

Our results suggest that the mental health consequences associated with the COVID-19 pandemic are significant to children, youth, and their parents, including as a cost within the healthcare system^65^. During a health crisis that requires social lockdown, many parents spend increased time at home and thus have the ability to intentionally monitor not only their own mental health but also their child or youth’s mental health with enhanced sensitivity to observe symptoms of worsened mental health^58^. Within this context, it is critical that parents have the knowledge and support required to inquire about or identify mental health difficulties within their families, especially among children and youth. A routine check-in for many children and youth is the pediatrician or family physician’s office; additional at-home resources should be made available to families. The delivery of mental health resources urgently requires expansion to increase scalability while prioritizing equitable access (e.g., multiple languages and appointment time periods, affordable care) across diverse populations^66^.

Sociologists have applied an illness behavior perspective to the study of family burden, identifying that impacts on mental health for one family member have far-reaching effects on other family members as individuals and as members of a social system^67,68^. Concerns related to COVID-19 are not universally detrimental as, for example, individuals may experience a reduction in work-life or school-life conflict^69^ or in stress from potential high-risk COVID-19 exposures at schools or in the workplace^70,71^. The relative capacity of unemployed versus employed parents to spend more time with their children or youth in place of work-life conflicts^72^, receipt of emergency financial benefits that alleviated financial constraint due to job loss^73^, and that emergency financial benefits may have exceeded wages from low-paying employments^74^ may further protect against detrimental impacts. Additional dyadic quantitative and qualitative studies are required to better understand and uncover possible mechanisms by which social-economic characteristics are associated with mental health during periods of health crisis.

Researchers, policymakers and public health practitioners have a unique opportunity to address disparities in mental health knowledge acquisition among children and youth^75,76^. Most parents in our study were aware of the mental health threat posed by the COVID-19 pandemic^77,78^; however, we found an overall lack of confidence in mental health knowledge acquisition among children and youth including limited vetting of the knowledge they do have. For optimal uptake and sustained memory of knowledge, mental health literacy campaigns should be made in partnership with youth^79–81^ and tailored to their preferences^82^. Our findings highlight the importance of healthcare professionals as conduits for accurate and valid information^83–86^. School or community healthcare education may be an accessible method for healthcare professionals to promote mental health to students^87^ as children and youth are less likely to exist within these pre-established relationships^80^. Child and youth friendly practices can be improved by including empathetic and friendly staff^87^, offering flexible hours^79^, using appropriate communication skills^82^ such as providing complex information in engaging, easy-to-understand language^88^, and conveying health information that supports their learning, so children and youth can be empowered to decide their own health views^89,90^. Mental health support delivered virtually may be a low-risk approach for increased access to care, especially for marginalized youth (e.g., racialized youth) and youth living in areas with limited access to in-person services^91,92^.

Improving mental health outcomes for children and youth in the recovery from the COVID-19 pandemic should be a research priority as we prepare for future pandemics. The rapid proliferation of child and youth mental health research during the COVID-19 pandemic impacted the quality of the execution of these studies that have been criticized for opting for timeliness at the expense of methodological quality^93,94^. Nonprobability or convenience samples were used in studies estimating prevalence of mental health disorders during the pandemic, which increases the likelihood of reporting bias^93^. We employed a nationally representative, multi-informant survey to explore changes in mental health symptoms from before to after the pandemic. Population-based cohort studies with longitudinal follow-up to monitor changes over time should be prioritized in future research.

### Limitations

We used a cross-sectional survey design that allowed data collection from a large and representative sample of the Canadian population. However, our results have limited longitudinal applicability; we queried respondents to report retrospectively on perceived changes in mental health symptoms during the COVID-19 pandemic and cannot generalize our findings to changes in mental health symptoms that occurred in the post-pandemic period. We also relied on a volunteer panel (Leger’s LEO panel) to recruit participants for compensation that may have introduced recruitment bias. Our survey was deployed online in English and French languages—Canada’s two official languages—that excluded individuals without internet access or those who read and write exclusively in other languages (~ 9% and ~ 2% of the Canadian population, respectively)^95^.

## Conclusions

Data from this cross-national survey highlights the importance of social factors in understanding pandemic-related changes to mental health symptoms of children, youth, and their families. Understanding patterns of mental health and factors associated with changes are essential to ensure that services match the needs of the population served. Ongoing surveillance of mental health among children, youth, and their families, as well as system-level planning are important to facilitate effective mental health campaigns and efficient use and development of mental health resources in periods of health crisis.

## Supplementary Information

Supplementary Information.

## Data Availability

The data are not publicly available due to them containing semi-identifiable information that could compromise research participant privacy. Additional summary tables of count data are available from the corresponding author upon reasonable request.
