# Supplementary material for: A muti-informant national survey on the impact of COVID-19 on mental health symptoms of parent–child dyads in Canada
Source: Sci Rep. 2023 May 17;13:7972. doi: 10.1038/s41598-023-34544-7 (PMC10189235; doi:10.1038/s41598-023-34544-7)
Supplement: Supplementary file 1 — Supplementary Information. [file 41598_2023_34544_MOESM1_ESM.docx]

**Supplemental Table 1.** CHERRIES

| ***Item Category*** | ***Checklist Item*** | ***Explanation*** | ***Page*** |
| --- | --- | --- | --- |
| **Design** | | | |
|  | *Describe survey design* | Describe target population, sample frame. Is the sample a convenience sample? | 8 |
| **IRB (Institutional Review Board) approval and informed consent process** | | | |
|  | *IRB approval* | Mention whether the study has been approved by an IRB. | 9 |
|  | *Informed consent* | Describe the informed consent process. Where were the participants told the length of time of the survey, which data were stored and where and for how long, who the investigator was, and the purpose of the study? | 9 |
|  | *Data protection* | If any personal information was collected or stored, describe what mechanisms were used to protect unauthorized access. | 8 |
| **Development and pre-testing** | | | |
|  | *Development and testing* | State how the survey was developed, including whether the usability and technical functionality of the electronic questionnaire had been tested before fielding the questionnaire. | 8 |
| **Recruitment process and description of the sample having access to the questionnaire** | | | |
|  | *Open survey versus closed survey* | An “open survey” is a survey open for each visitor of a site, while a closed survey is only open to a sample which the investigator knows (password-protected survey). | 9 |
|  | *Contact mode* | Indicate whether or not the initial contact with the potential participants was made on the Internet. (Investigators may also send out questionnaires by mail and allow for Web-based data entry.) | 9 |
|  | *Advertising the survey* | How/where was the survey announced or advertised? Some examples are offline media (newspapers), or online (mailing lists – If yes, which ones?) or banner ads (Where were these banner ads posted and what did they look like?). It is important to know the wording of the announcement as it will heavily influence who chooses to participate. Ideally the survey announcement should be published as an appendix. | 9 |
| **Survey administration** | | | |
|  | *Web/E-mail* | State the type of e-survey (eg, sent out through e-mail). If it is an e-mail survey, were the responses entered manually into a database, or was there an automatic method for capturing responses? | 9 |
|  | *Context* | Describe the Web site (for mailing list/newsgroup) in which the survey was posted. What is the Web site about, who is visiting it, what are visitors normally looking for? Discuss to what degree the content of the Web site could pre-select the sample or influence the results. | 9 |
|  | *Mandatory/voluntary* | Was it a mandatory survey to be filled in by every visitor who wanted to enter the Web site, or was it a voluntary survey? | 9 |
|  | *Incentives* | Were any incentives offered (eg, monetary, or non-monetary incentives? | 8 |
|  | *Time/Date* | In what timeframe were the data collected? | 8 |
|  | *Randomization of items or questionnaires* | To prevent biases items can be randomized or alternated. | 9 |
|  | *Adaptive questioning* | Use adaptive questioning (certain items, or only conditionally displayed based on responses to other items) to reduce number and complexity of the questions. | 9 |
|  | *Number of Items* | What was the number of questionnaire items per page? | 8 |
|  | *Completeness check* | It is technically possible to do consistency or completeness checks before the questionnaire is submitted. Was this done, and if “yes”, how? An alternative is to check for completeness after the questionnaire has been submitted. If this has been done, it should be reported. All items should provide a non-response option such as “not applicable” or “rather not say”, and selection of one response option should be enforced. | 9 |
|  | *Review step* | State whether respondents were able to review and change their answers (eg, through a Back button or a Review step which displays a summary of the responses and asks the respondents if they are correct). | 9 |
| **Response rates** | | | |
|  | *Unique site visitor* | If you provide view rates or participation rates, you need to define how you determined a unique visitor. There are different techniques available, based on IP addresses or cookies or both. | N/A |
|  | *View rate (Ratio of unique survey visitors/unique site visitors)* | Requires counting unique visitors to the first page of the survey, divided by the number of unique site visitors (not page views!). It is not unusual to have view rates of less than 0.1 % if the survey is voluntary. | N/A |
|  | *Participation rate (Ratio of unique visitors who agreed to participate/unique first survey page visitors)* | Count the unique number of people who filled in the first survey page (or agreed to participate, for example by checking a checkbox), divided by visitors who visit the first page of the survey (or the informed consents page, if present). This can also be called “recruitment” rate. | N/A |
|  | *Completion rate (Ratio of users who finished the survey/users who agreed to participate)* | The number of people submitting the last questionnaire page, divided by the number of people who agreed to participate (or submitted the first survey page). This is only relevant if there is a separate “informed consent” page or if the survey goes over several pages. This is a measure for attrition. Note that “completion” can involve leaving questionnaire items blank. This is not a measure for how completely questionnaires were filled in. (If you need a measure for this, use the word “completeness rate”.) | 9 |
| **Preventing multiple entries from the same individual** | | | |
|  | *Cookies used* | Indicate whether cookies were used to assign a unique user identifier to each client computer. If so, mention the page on which the cookie was set and read, and how long the cookie was valid. Were duplicate entries avoided by preventing users access to the survey twice; or were duplicate database entries having the same user ID eliminated before analysis? In the latter case, which entries were kept for analysis (eg, first or the most recent)? | 8, 9 |
|  | *IP check* | Indicate whether the IP address of the client computer was used to identify potential duplicate entries from the same user. If so, mention the period of time for which no two entries from the same IP address were allowed (eg, 24 hours). Were duplicate entries avoided by preventing users with the same IP address access to the survey twice; or were duplicate database entries having the same IP address within a given period of time eliminated before analysis? If the latter, which entries were kept for analysis (eg, the first entry or the most recent)? | N/A |
|  | *Registration* | In “closed” (non-open) surveys, users need to login first and it is easier to prevent duplicate entries from the same user. Describe how this was done. For example, was the survey never displayed a second time once the user had filled it in, or was the username stored together with the survey results and later eliminated? If the latter, which entries were kept for analysis (eg, the first entry or the most recent)? | 8, 9 |
| **Analysis** | | | |
|  | *Handling of incomplete questionnaires* | Were only completed questionnaires analyzed? Were questionnaires which terminated early (where, for example, users did not go through all questionnaire pages) also analyzed? | 10 |
|  | *Statistical correction* | Indicate whether any methods such as weighting of items or propensity scores have been used to adjust for the non-representative sample; if so, please describe the methods. | 10 |

**Supplemental Table 2.** The Partnership for Maternal, Newborn & Child Health and the World Health Organization of the United Nations H6+ Technical Working Group on Adolescent Health and Well-Being consensus framework^1^

| **Domain** | **Subdomains** |
| --- | --- |
| *(1) Good health and optimum nutrition* | • Physical health and capacities. • Mental health and capacities. • Optimal nutritional status and diet |
| *(2) Connectedness, positive values, and contribution to society* | • Connectedness: Is part of positive social and cultural networks and has positive, meaningful relationships with others, including family, peers, and, where relevant, teachers and employers. • Valued and respected by others and accepted as part of the community. • Attitudes: Responsible, caring, and has respect for others. Has a sense of ethics, integrity, and morality. • Interpersonal skills: Empathy, friendship skills, and sensitivity. • Activity: Socially, culturally, and civically active. • Change and development: Equipped to contribute to change and development in their own lives and/or in their communities. |
| *(3) Safety and a supportive environment* | • Safety: Emotional and physical safety. • Material conditions in the physical environment are met. • Equity: Treated fairly and have an equal chance in life. • Equality: Equal distribution of power, resources, rights, and opportunities for all. • Nondiscrimination. • Privacy. • Responsive: Enriching the opportunities available to the adolescent. |
| *(4) Learning, competence, education, skills, and employability* | • Learning: Has the commitment to, and motivation for, continual learning. • Education. • Resources, life skills, and competencies: Has the necessary cognitive, social, creative, and emotional resources, skills (life/decision-making) and competencies to thrive, including knowing their rights and how to claim them, and how to plan and make choices. • Skills: Acquisition of technical, vocational, business, and creative skills to be able to take advantage of current or future economic, cultural, and social opportunities. • Employability. • Confidence that they can do things well. |
| *(5) Agency and resilience* | • Agency: Has self-esteem, a sense of agency and of being empowered to make meaningful choices and to influence their social, political, and material environment and has the capacity for self-expression and self-direction appropriate to their evolving capacities and stage of development. • Identity: Feels comfortable in their own self and with their identity(s), including their physical, cultural, social, sexual, and gender identity. • Purpose: Has a sense of purpose, desire to succeed, and optimism about the future. • Resilience: Equipped to handle adversities both now and in the future, in a way that is appropriate to their evolving capacities and stage of development. • Fulfilment: Feels that they are fulfilling their potential now and that they will be able to do so in the future. |

^1^Ross DA, Hinton R, Melles-Brewer M, et al. Adolescent Well-Being: A Definition and Conceptual Framework. J Adolesc Health. 2020;67(4):472-476.

**Supplemental Table 3.** Age-tailored, operational definitions for mental health symptoms provided in the online survey

| **Symptoms** | **Definitions** |
| --- | --- |
| *Anxiety* | **Parent and Youth:** An emotion characterized by feelings of tension, worried thoughts, and physical changes like increased blood pressure.  **Child:** Having thoughts or feelings that can be very scary or that worry you. |
| *Hyperactivity* | **Parent and Youth:** Behaviours that refer to constant activity, impulsiveness, and similar behaviors such as fidgeting.  **Child:** Feeling like you always need to be moving or having trouble sitting still. |
| *Inattention/Fatigue* | **Parent and Youth:** Behaviors characterized by inability to focus, high levels of distractibility, forgetfulness, and poor organization and planning.  **Child:** When you can’t seem to focus on what you should be doing or forgetting what you need to do. |
| *Irritability* | **Parent and Youth:** Irritability involves feelings of anger or frustration that often arise over even the smallest of things.  **Child:** Being annoyed easily at things going on around you. |
| *Mood* | **Parent and Youth:** Poor mood might be when you feel empty, helpless, or inadequate, have low self-esteem or loss of interest in usual activities.  **Child:** Feeling sad or down or not wanting to do the things that you like to do. |
| *Obsessions/Compulsions* | **Parent and Youth:** Obsessions are unwanted, intrusive thoughts, images, or urges that trigger intensely distressing feelings. Compulsions are behaviors an individual engages in to attempt to get rid of the obsessions and/or decrease distress.  **Child:** Having thoughts or feelings that make you feel extremely uncomfortable and doing things to get rid of those same thoughts or feelings. |

Respondents were asked, “Compared to the time before the COVID-19 pandemic, how is your [mental health domain].”

**Supplemental Table 4.** Mental Health Impacts of 933 Child/Youth-Adult Dyad Survey Participants in the COVID-19 Pandemic

| **Mental Health Domains** | **Likert-type Values** | **Parent**  **N=450** | | **Youth**  **N=450** | | **P-value** | **Parent**  **N=483** | | **Child**  **N=483** | | **P-value** |
| --- | --- | --- | --- | --- | --- | --- | --- | --- | --- | --- | --- |
|  |  | **n** | **%** | **n** | **%** |  | **n** | **%** | **n** | **%** |  |
| Mood | 1: A lot worse | 44 | 9.8% | 27 | 6.0% | <0.001 | 54 | 11.2% | 22 | 4.6% | <0.001 |
|  | 2: A little worse | 126 | 28.0% | 98 | 21.8% |  | 149 | 30.8% | 127 | 26.3% |  |
|  | 3: The same | 216 | 48.0% | 263 | 58.4% |  | 209 | 43.3% | 258 | 53.4% |  |
|  | 4: A little better | 47 | 10.4% | 48 | 10.7% |  | 46 | 9.5% | 52 | 10.8% |  |
|  | 5: A lot better | 17 | 3.8% | 14 | 3.1% |  | 25 | 5.2% | 24 | 5.0% |  |
| Anxiety | 1: A lot worse | 68 | 15.1% | 44 | 9.8% | 0.006 | 82 | 17.0% | 44 | 9.1% | <0.001 |
|  | 2: A little worse | 136 | 30.2% | 123 | 27.3% |  | 150 | 31.1% | 137 | 28.4% |  |
|  | 3: The same | 196 | 43.6% | 239 | 53.1% |  | 192 | 39.8% | 241 | 49.9% |  |
|  | 4: A little better | 35 | 7.8% | 31 | 6.9% |  | 38 | 7.9% | 36 | 7.5% |  |
|  | 5: A lot better | 16 | 3.6% | 13 | 2.9% |  | 21 | 4.3% | 25 | 5.2% |  |
| Irritability | 1: A lot worse | 49 | 10.9% | 35 | 7.8% | 0.32 | 67 | 13.9% | 32 | 6.6% | <0.001 |
|  | 2: A little worse | 121 | 26.9% | 125 | 27.8% |  | 157 | 32.5% | 128 | 26.5% |  |
|  | 3: The same | 229 | 50.9% | 246 | 54.7% |  | 194 | 40.2% | 272 | 56.3% |  |
|  | 4: A little better | 35 | 7.8% | 30 | 6.7% |  | 43 | 8.9% | 32 | 6.6% |  |
|  | 5: A lot better | 16 | 3.6% | 14 | 3.1% |  | 22 | 4.6% | 19 | 3.9% |  |
| Inattention/fatigue | 1: A lot worse | 58 | 12.9% | 32 | 7.1% | 0.028 | 79 | 16.4% | 37 | 7.7% | <0.001 |
|  | 2: A little worse | 109 | 24.2% | 114 | 25.3% |  | 142 | 29.4% | 119 | 24.6% |  |
|  | 3: The same | 227 | 50.4% | 256 | 56.9% |  | 210 | 43.5% | 266 | 55.1% |  |
|  | 4: A little better | 43 | 9.6% | 35 | 7.8% |  | 31 | 6.4% | 39 | 8.1% |  |
|  | 5: A lot better | 13 | 2.9% | 13 | 2.9% |  | 21 | 4.3% | 22 | 4.6% |  |
| Hyperactivity | 1: A lot worse | 23 | 5.1% | 18 | 4.0% | 0.67 | 23 | 4.8% | 18 | 3.7% | 0.22 |
|  | 2: A little worse | 53 | 11.8% | 63 | 14.0% |  | 53 | 11.0% | 65 | 13.5% |  |
|  | 3: The same | 322 | 71.6% | 320 | 71.1% |  | 349 | 72.3% | 325 | 67.3% |  |
|  | 4: A little better | 40 | 8.9% | 32 | 7.1% |  | 42 | 8.7% | 49 | 10.1% |  |
|  | 5: A lot better | 12 | 2.7% | 17 | 3.8% |  | 16 | 3.3% | 26 | 5.4% | 0.06 |
| Obsessions/compulsions | 1: A lot worse | 26 | 5.8% | 19 | 4.2% | 0.66 | 37 | 7.7% | 24 | 5.0% |  |
|  | 2: A little worse | 66 | 14.7% | 69 | 15.3% |  | 77 | 15.9% | 77 | 15.9% |  |
|  | 3: The same | 309 | 68.7% | 320 | 71.1% |  | 317 | 65.6% | 323 | 66.9% |  |
|  | 4: A little better | 33 | 7.3% | 26 | 5.8% |  | 36 | 7.5% | 40 | 8.3% |  |
|  | 5: A lot better | 16 | 3.6% | 16 | 3.6% |  | 16 | 3.3% | 19 | 3.9% |  |

Respondents were asked, “Compared to the time before the COVID-19 pandemic, how is your [mental health domain.” P-values obtained using Wilcoxon signed rank test, taking into the paired nature of the comparison. This means 450 parents of youth were compared to their youth, and the 483 parents of children were compared to their children. Respondents who did not provide an answer were excluded from analyses.

**Supplemental Table 5.** Mental Health Literacy of 933 Child/Youth-Adult Dyad Survey Participants in the COVID-19 Pandemic

| **Question** | **Likert-type Values** | **Parent**  **N=450** | | **Youth**  **N=450** | | **P-value** | **Parent**  **N=483** | | **Child**  **N=483** | | **P-value** |
| --- | --- | --- | --- | --- | --- | --- | --- | --- | --- | --- | --- |
| I compare health information from different sources. | 1: Completely disagree | 55 | 12.2% | 46 | 10.2% | <0.001 | 65 | 13.5% | 45 | 9.3% | <0.001 |
|  | 2: Somewhat disagree | 96 | 21.3% | 85 | 18.9% |  | 98 | 20.3% | 88 | 18.2% |  |
|  | 3: Unsure/no opinion | 45 | 10.0% | 142 | 31.6% |  | 53 | 11.0% | 190 | 39.3% |  |
|  | 4: Somewhat agree | 176 | 39.1% | 143 | 31.8% |  | 174 | 36.0% | 120 | 24.8% |  |
|  | 5: Completely agree | 78 | 17.3% | 34 | 7.6% |  | 93 | 19.3% | 40 | 8.3% |  |
| When I hear about or read about new health information I verify if it is true or not. | 1: Completely disagree | 59 | 13.1% | 34 | 7.6% | 0.013 | 72 | 14.9% | 37 | 7.7% | 0.045 |
|  | 2: Somewhat disagree | 84 | 18.7% | 97 | 21.6% |  | 86 | 17.8% | 91 | 18.8% |  |
|  | 3: Unsure/no opinion | 46 | 10.2% | 109 | 24.2% |  | 46 | 9.5% | 133 | 27.5% |  |
|  | 4: Somewhat agree | 151 | 33.6% | 155 | 34.4% |  | 158 | 32.7% | 170 | 35.2% |  |
|  | 5: Completely agree | 110 | 24.4% | 55 | 12.2% |  | 121 | 25.1% | 52 | 10.8% |  |
| I decide what health information is best for me. | 1: Completely disagree | 35 | 7.8% | 27 | 6.0% | <0.001 | 36 | 7.5% | 33 | 6.8% | <0.001 |
|  | 2: Somewhat disagree | 72 | 16.0% | 95 | 21.1% |  | 71 | 14.7% | 100 | 20.7% |  |
|  | 3: Unsure/no opinion | 55 | 12.2% | 106 | 23.6% |  | 66 | 13.7% | 134 | 27.7% |  |
|  | 4: Somewhat agree | 168 | 37.3% | 151 | 33.6% |  | 194 | 40.2% | 159 | 32.9% |  |
|  | 5: Completely agree | 120 | 26.7% | 71 | 15.8% |  | 116 | 24.0% | 57 | 11.8% |  |
| I can identify if health information is relevant to me or not. | 1: Completely disagree | 66 | 14.7% | 35 | 7.8% | <0.001 | 55 | 11.4% | 30 | 6.2% | <0.001 |
|  | 2: Somewhat disagree | 86 | 19.1% | 109 | 24.2% |  | 90 | 18.6% | 86 | 17.8% |  |
|  | 3: Unsure/no opinion | 39 | 8.7% | 116 | 25.8% |  | 42 | 8.7% | 171 | 35.4% |  |
|  | 4: Somewhat agree | 151 | 33.6% | 123 | 27.3% |  | 190 | 39.3% | 155 | 32.1% |  |
|  | 5: Completely agree | 108 | 24.0% | 67 | 14.9% |  | 106 | 21.9% | 41 | 8.5% |  |
| I ask a health professional about the quality of information I find. | 1: Completely disagree | 55 | 12.2% | 51 | 11.3% | 0.008 | 55 | 11.4% | 57 | 11.8% | 0.024 |
|  | 2: Somewhat disagree | 99 | 22.0% | 97 | 21.6% |  | 100 | 20.7% | 94 | 19.5% |  |
|  | 3: Unsure/no opinion | 68 | 15.1% | 136 | 30.2% |  | 89 | 18.4% | 152 | 31.5% |  |
|  | 4: Somewhat agree | 152 | 33.8% | 119 | 26.4% |  | 167 | 34.6% | 122 | 25.3% |  |
|  | 5: Completely agree | 76 | 16.9% | 47 | 10.4% |  | 72 | 14.9% | 58 | 12.0% |  |

P-values obtained using Wilcoxon signed rank test, taking into the paired nature of the comparison. This means 450 parents of youth were compared to their youth, and the 483 parents of children were compared to their children. Respondents who did not provide an answer were excluded from analyses.

**Supplemental Figure 1.** Mental Health Impacts of 933 Adult-Child (top) or Youth (bottom) Dyad Survey Participants in the COVID-19 Pandemic, by Financial Stability

**Supplemental Figure 2.** Mental Health Impacts of 933 Adult-Child (top) or Youth (bottom) Dyad Survey Participants in the COVID-19 Pandemic, by Housing Stability

**Supplemental Figure 3.** Mental Health Impacts of 933 Adult-Child (top) or Youth (bottom) Dyad Survey Participants in the COVID-19 Pandemic, by Self-identifying as Living with a Disability

**Supplemental Figure 4.** Mental Health Impacts of 933 Adult-Child (top) or Youth (bottom) Dyad Survey Participants in the COVID-19 Pandemic, by Ethnicity

**Supplemental Figure 5.** Mental Health Impacts of 933 Adult-Child (left) or Youth (right) Dyad Survey Participants in the COVID-19 Pandemic, by Canadian Residence

**Supplemental Figure 6.** Mental Health Impacts of 933 Adult-Child (left) or Youth (right) Dyad Survey Participants in the COVID-19 Pandemic, by Gender

**Supplemental Figure 7.** Mental Health Impacts of 933 Adult-Child (left) or Youth (right) Dyad Survey Participants in the COVID-19 Pandemic, by Geographical Location

**Supplemental Figure 8.** Mental Health Impacts of 933 Adult-Child (left) or Youth (right) Dyad Survey Participants in the COVID-19 Pandemic, by Employment Status

**Supplemental Figure 9.** Mental Health Impacts of 933 Adult-Child (left) or Youth (right) Dyad Survey Participants in the COVID-19 Pandemic, by Household Size
